# Higher COVID-19 Vaccination Rates Are Associated with Lower COVID-19 Mortality: A Global Analysis

**DOI:** 10.3390/vaccines11010074

**Published:** 2022-12-28

**Authors:** Ilir Hoxha, Riaz Agahi, Altina Bimbashi, Mrika Aliu, Lul Raka, Ilirjana Bajraktari, Petrit Beqiri, Lisa V. Adams

**Affiliations:** 1 The Dartmouth Institute for Health Policy and Clinical Practice, Geisel School of Medicine at Dartmouth, Lebanon, NH 03766, USA; 2 Research Unit, Heimerer College, 10000 Prishtina, Kosovo; 3 Evidence Synthesis Group, 10000 Prishtina, Kosovo; 4 Faculty of Medicine, University of Prishtina, 10000 Prishtina, Kosovo; 5 European Group on Health Care Delivery, 55305 Jonkoping, Sweden; 6 Institute for Health and Nursing Science, Faculty of Medicine, Martin Luther University Halle-Wittenberg, 06108 Halle (Saale), Germany; 7 Centre for Global Health Equity, Geisel School of Medicine at Dartmouth, Hanover, NH 03755, USA

**Keywords:** global health, coronavirus, mortality, vaccinations

## Abstract

Mass vaccination initiatives are underway worldwide, and a considerable percentage of the world’s population is now vaccinated. This study examined the association of COVID-19 deaths per 1000 cases with a fully vaccinated population. The global median deaths per 1000 cases were 15.68 (IQR 9.84, 25.87) after 6 months of vaccinations and 11.96 (IQR 6.08, 20.63) after 12 months. Across 164 countries, we found significant variations in vaccination levels of populations, booster doses, and mortality, with higher vaccine coverage and lower mortality in high-income countries. Several regression models were performed to test the association between vaccination and COVID-19 mortality. Control variables were used to account for confounding variables. A 10-percentage-point increase in vaccination was associated with an 18.1% decrease in mortality after 6 months (95%CI, 7.4–28.8%) and a 16.8% decrease after 12 months (95%CI, 6.9–26.7%). A 10-percentage-point increase in booster vaccination rates was associated with a 33.1% decrease in COVID-19 mortality (95%CI, 16.0–50.2%). This relationship is present in most analyses by country income groups with variations in the effect size. Efforts are needed to reduce vaccine hesitancy while ensuring suitable infrastructure and supply to enable all countries to increase their vaccination rates.

## 1. Introduction

COVID-19 prevalence is approaching 630 million cases globally, with over 6.5 million deaths [1] and at least 6% of cases resulting in long COVID symptoms [2]. This has created a significant strain on health services [3] and considerable ongoing health problems. Low-income populations [4] and vulnerable groups, such as the elderly or those with comorbidities, have been particularly negatively affected [5].

COVID-19 vaccinations are now underway globally, and at the time of writing, over 12.8 billion doses have been administered [1]. The benefits [6] of vaccines in preventing cases are well established, including for vulnerable groups [7]; however, questions remain, particularly in light of potential adverse reactions. Such reactions are generally mild and brief, but rare cases of more severe reactions have been reported [8]. Despite the positive results obtained with vaccines and concerted public health efforts to administer vaccination programs, there remains considerable skepticism around vaccination [9]. Therefore, it is important that strategies are employed to build trust in the wider population [10] by communicating the benefits of COVID-19 vaccination at the population level and addressing misinformation [11].

Questions have also arisen concerning new variants and breakthrough infections [12]. Studies have shown vaccination efficacy against some variants, with especially high activity against the alpha variant and lower activity against other variants, particularly the beta variant [13] and the delta variant [14,15]. New vaccines, such as a vaccine based on inactivated SARS-CoV-2, are in clinical trials and have been used in some emergency circumstances [16]. Emphasis is now being placed on the use of booster shots to enhance and lengthen immunity [17] as the levels of immunity for some vaccines seem to wane over the course of 6 months [18].

Since the beginning of the pandemic, there has been considerable effort to contain the virus, including restrictive measures from governments [19] as well as efforts from researchers to understand the pandemic’s evolution and control. Some key areas of inquiry have been potential symptoms [20] and therapies [21], the effect of the pandemic on lifestyle factors such as diet [22], understanding the mechanism of transmission and human–pathogen interactions [23,24,25], and perhaps most crucially, vaccines.

Vaccination research is ongoing, for example, the efficacy of new vaccinations based on mRNA, protein subunits, or viral vectors [26]. The effects of existing vaccinations have also been examined.For example, a systematic review by Marra and coworkers reported that the likelihood of post-COVID-19 symptoms was significantly reduced in those who had received at least one vaccine dose [27]. Another important area of examination is the effectiveness of vaccinations against COVID-19 variants [13,28]. It has been reported that vaccinations currently used have lower effectiveness against the Omicron variant in comparison to other variants. Nevertheless, there is still a strong protective effect. Therefore, there is a need for the development of new vaccines, but this does not undermine the overwhelming advantages of currently available vaccines [29].

Given the salutary effects of vaccination, the geographical spread of vaccines and sociopolitical factors affecting their availability are highly relevant areas of research. It has been reported that vaccination rates vary not only between countries but across ethnicity and income groups within a country [30]. Global variation in vaccines has been reported to have a significant toll on population health in terms of cases and deaths [31]. Efforts towards tackling vaccine inequity should be based on vaccine empathy, i.e., the desire to ameliorate the challenges associated with low vaccinations in other countries, or vaccine diplomacy, i.e., the use of vaccines as a tool to improve relations between countries [32].

Another important topic that can be addressed by research is the prevalence and causes of vaccine hesitancy. Several factors seem to be pertinent in this case. A lack of trust in medical and government institutions influences decisions to avoid vaccination [33]. Similarly, a lack of confidence in the vaccines themselves, in terms of potential side effects or lack of efficacy, has also been identified as a barrier to vaccination [34]. Tackling misinformation is crucial to implementing mass vaccination, and in a study carried out with students in Germany, it was found that students who felt better informed about the scientific underpinning of government decisions and the vaccines, in general, were more likely to be vaccinated [35]. Methods based on social media and other technology can have value not only in countering misinformation but in addressing unmet needs as a result of the pandemic [36,37].

Many studies have examined the immunological effects of vaccinations, but few have examined how vaccination rates have affected COVID-19 severity at the country level. An ecological study design can give a broad view of trends in health and health system performance, for example, trends in NCD mortality or disease prevalence [38,39]. As such, our study explored the impact of COVID-19 vaccination coverage on the outcomes of COVID-19 cases at the population level, in particular, deaths per 1000 cases.

## 2. Materials and Methods

### 2.1. Data Sources

We used data from several sources, including the Global Health Expenditure Database and Global Health Observatory data from the World Health Organization (WHO) [40], as well as World Bank Open Data [20]. Data regarding COVID-19 related outcomes, i.e., cases, deaths, and percent of the vaccinated population, were obtained from WHO’s publicly available COVID-19 dashboard [1]. The vaccination data provided by the WHO includes vaccination with any of the WHO Emergency Use Listing vaccines. We used data available as of 20 September 2022. For other variables, we used data from the most recent year where data was available.

### 2.2. Outcome Variables

The primary outcome measure was COVID-19 mortality, defined as the number of deaths per 1000 COVID-19 cases. We used two separate variables for mortality, i.e., the death per case rate 6 months and 12 months after beginning the first vaccinations in each country. These variables were created by dividing the cumulative number of deaths by the cumulative number of cases in a country within the specified period, then multiplying by 1000. Our secondary outcome measure was the death per case ratio by country income groups. Accordingly, data for cases and deaths were selected to match the first 6 months or 12 months of vaccinations in each country. We expected that countries with higher vaccination rates would have fewer deaths per cases.

### 2.3. Exposure Variables

Overall, three exposure (independent) variables were included in the analysis. These included the percentage of fully vaccinated individuals in a country in the first 6 and 12 months of vaccinations and the percentage of the population who had received a booster dose within the first 12 months of vaccine implementation. For each country, we identified the date when vaccinations commenced (according to the WHO COVID-19 Dashboard) [1]. We then added a two week period, which is the amount of time required for vaccinations to take effect [6]. We then compiled vaccination rates for each included country 6 months and 12 months after the beginning of vaccinations. This provided a more accurate assessment of the effect of vaccination levels than simply applying the date of first vaccinations globally to every country. 

### 2.4. Control Variables

We expected that population factors such as comorbidities or the country’s Gross Domestic Product (GDP) would significantly impact the results. We, therefore, integrated several country-level variables into our analytical models. We included GDP per capita to account for economic differences and the population’s median age, which was taken from the latest data from the UN Population Division. Global values vary considerably and have been reported as a significant variable in COVID-19 mortality [5]. In addition, we included the percent prevalence of obesity (defined as BMI ≥ 30) as another control variable, given its association with unfavorable COVID-19 outcomes [5], and overall non-communicable disease (NCD) mortality per 100,000 to account for comorbidities.

### 2.5. Statistical Analysis

For analytical purposes, we grouped the sample of countries into a total sample and subsets by income classification (i.e., high, upper-middle income, lower-middle income, and low income countries). We examined the countries by income groups to account for structural differences that correlate to the income status of countries. To express the distribution of continuous data, we calculated the mean and standard deviation for normally distributed data. Normal distribution was determined using a histogram, QQ plot, and Shapiro–Wilk test. We calculated the median and interquartile range for continuous data without normal distribution. Then global distribution of death case rates and vaccination percentages was presented graphically using maps.

Our main analysis examined the association of country death case ratio with vaccination rates. We performed regression analysis for the total sample and each subset of countries. Each country constituted a distinct observation. We used ordinary least squares (OLS) to estimate the β of the log-linear model log Y = α + β X, where Y is the dependent variable of interest in each country (i.e., death/case ratio) and X represents the corresponding vaccination coverage of the country. We opted for this model due to the non-normal distribution of the primary outcome variables used in this model (i.e., death case ratio). We examined two models for each outcome and exposure of interest. (1) a simple bivariate model and (2) a model adjusting for GDP per capita, the median age of the country’s population, the prevalence of obesity among adults, and the age-standardized NCD mortality rate. We added these variables to each model to reduce potential confounding. We used multiple imputations with a chained equations algorithm to address missing data on some of the control variables. All analyses were conducted using Stata BE 17, (Stata Corp., College Station, TX, USA).

## 3. Results

**Figure 1:**
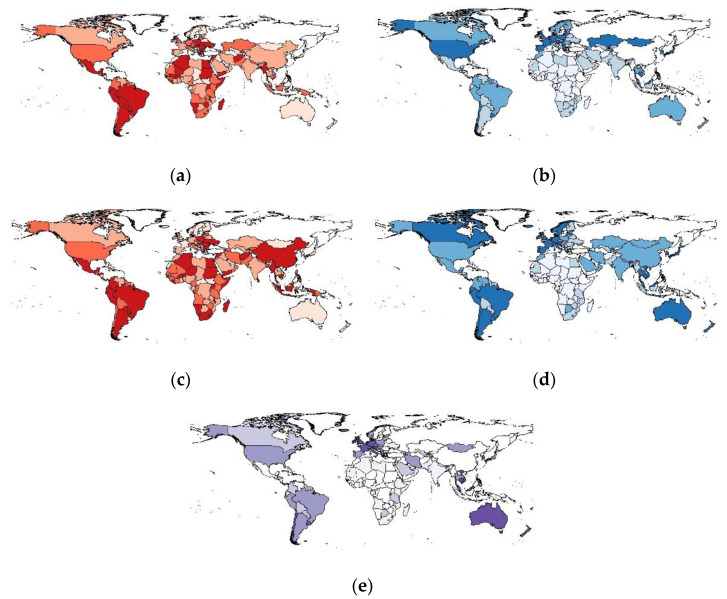
Choropleth maps showing global variation in COVID-19-related variables: (**a**) Deaths per 1000 cases (6 months after vaccine implementation); (**b**) % Fully Vaccinated Population (6 months after vaccine implementation); (**c**) Deaths per 1000 cases (12 months after vaccine implementation); (**d**) % Fully Vaccinated Population (12 months after vaccine implementation); (**e**) % of population to have received booster doses (12 months after vaccine implementation). The maps represent the classification of countries by the quartiles they belong to. Countries be-longing to the upper quartiles are marked with a darker color. Countries belonging to lower quartiles are marked with a lighter color. Countries with no data are marked with white color.

We visualized this study’s primary exposure and outcome variables using choropleth world maps (Figure 1). Maps were generated for deaths per 1000 cases for 6 months (Figure 1a) and 12 months (Figure 1c) after the beginning of vaccine implementation. We also generated maps to show the speed of vaccine rollout by examining the percentage of the fully vaccinated population after 6 months (Figure 1b) and 12 months (Figure 1d), in addition to the percentage of the population in each country who had received a booster dose (Figure 1e). These maps demonstrate the variation in mortality and vaccine rollout. In the case of booster doses, it is clear that several countries have made significant progress, while others have yet to implement booster doses.

We determined the median and IQR values for our key variables (Table 1). The overall median deaths per 1000 cases globally was 15.68 (IQR 9.84, 25.87) after 6 months of vaccinations and 11.96 (IQR 6.08, 20.63) after 12 months. For both variables, the median mortality rate increased with decreasing country income. For example, after 12 months, the median number of deaths was 6.37 (IQR 3.70, 13.63) in high-income countries and 21.81 (IQR 9.54, 34.79) in low income countries.

The overall median percentage of the fully vaccinated population after 6 and 12 months were 17.27 (IQR 4.50, 36.72) and 46.96 (IQR 23.07, 67.95), respectively, while the median population percentage who had received a booster dose after 12 months was 18.85 (IQR 5.56, 36.21). For all three variables, the median decreased progressively with decreasing country income classification. Notably, the median of the fully vaccinated population varies considerably between high-income countries (6 months—39.25, IQR 29.49, 48.94; 12 months—70.05, IQR 63.90, 77.06) and low income countries (6 months—1.19, IQR 0.50, 2.90; 12 months 10.68, IQR 5.15, 17.48). This was also observed for booster doses, where a median of 36.2% was observed for high-income countries, and a median of 0.29% was observed in low income countries.

Unadjusted and adjusted regression analyses of all countries showed a statistically significant inverse relationship between vaccination rates and COVID-19 mortality (Table 2). In the unadjusted analysis of data collected 6 months after the beginning of vaccine implementation, we see reduced deaths per 1000 cases with increased vaccination rates. The estimated value of β = −0.0160 means that a 10-percentage-point increase in vaccination rates was associated with a 16.0% decrease in COVID-19 mortality (95%CI, 7.9–24.1%). Adjusted analysis showed similar results. The estimated value of β = −0.0181 means that a 10-percentage-point increase in vaccination rates was associated with an 18.1% decrease in COVID-19 mortality (95%CI, 7.4–28.8%). The inverse relationship between vaccination rates and COVID-19 mortality was present in all analyses by income groups. The results, though, were statistically insignificant, except for adjusted analysis for the lower-middle income group. It is also worth noting variations in the effect size among income groups.

In the unadjusted analysis of data collected 12 months after the beginning of vaccine implementation, we again see reduced deaths per 1000 cases with increased vaccination rates. The estimated value of β = −0.0176 means that a 10-percentage-point increase in vaccination rates was associated with a 17.6% decrease in COVID-19 mortality (95%CI, 11.4–23.8%). Adjusted analysis showed similar results. The estimated value of β = −0.0168 means that a 10-percentage-point increase in vaccination rates was associated with a 16.8% decrease in COVID-19 mortality (95%CI, 6.9–26.7%). The inverse relationship between vaccination rates and COVID-19 mortality was present in all analyses by income groups except upper-middle income countries. The results were mainly statistically insignificant, except for unadjusted analysis for high and low income countries and adjusted analysis for the lower-middle income group. Again, there was also a considerable variation in the effect size among income groups.

Finally, in the unadjusted analysis of the percentage of the population who received a booster dose within 12 months of the first vaccinations, we see an even larger reduced number of deaths per 1000 cases with an increase in booster dose rates. The estimated value of β = −0.0310 means that a 10-percentage-point increase in booster rates was associated with a 31.0% decrease in COVID-19 mortality (95%CI, 19.9–42.1%). Adjusted analysis showed similar results. The estimated value of β = −0.0331 means that a 10-percentage-point increase in booster rates was associated with a 33.1% decrease in COVID-19 mortality (95%CI, 16.0–50.2%). The inverse relationship between booster rates and COVID-19 mortality was present in all analyses by income groups. The results, though, were statistically insignificant, except for the high income group. Again, it is also worth noting the variations in the effect size among income groups.

## 4. Discussion

### 4.1. Summary of Results

Our results clearly show lower COVID-19 mortality rates per 1000 cases for countries with higher COVID-19 vaccination coverage. Deaths per cases decreased following vaccine implementation [1]. This trend was observed in unadjusted and adjusted analyses controlling for several pertinent country-level variables. Similarly, adjusted analyses of the percentage of individuals who had received a booster dose showed that an increase in the percentage of booster doses was associated with significantly reduced COVID-19 deaths per 1000 cases. Significant inverse associations were found in most analyses in income groups. Another notable point is the contrast in effect estimates for countries of different income levels.

### 4.2. Context

Other studies have examined the effect of COVID-19 vaccinations on severe illness and hospitalizations [11], the risk for breakthrough infections [41], and the likelihood of contracting different variants of COVID-19 [13], and have observed considerable but reduced efficacy of vaccinations against variants. For example, a study in Ontario, Canada, reported between 82% and 95% vaccine efficacy against infections with variants and similar or higher effects against hospitalization [42]. Other studies have associated inter-country variation in COVID-19 fatality with health factors such as obesity and age [5]. The severity of the COVID-19 pandemic has also been linked to differences in policy [43]. The authors suggest that initial preparedness and swift implementation of measures may have influenced the performance of different countries.

Figueroa and coworkers reported that as late as June 2021, only 0.9% of the population of low-income counties were vaccinated, compared with 43% in high-income countries [44]. National and global efforts are needed to ensure all countries can achieve high population vaccination rates. Equitable global vaccine distribution is a critical factor in achieving this goal [44]. The ability to mitigate the effects of the pandemic and protect vulnerable members of society should not be limited only to higher-income countries. This is based not only on the obvious moral imperative and human rights-based approach but also because this is necessary to achieve global control of the pandemic [45].

Another important and interrelated family of measures for pandemic management is non-pharmaceutical interventions (NPIs). Studies have shown that NPIs are beneficial both from a health perspective and economically [46], and can significantly reduce COVID-19 incidence [47]. A study using data from Our World in Data demonstrated that NPIs had a protective effect on mortality. For example, during the pre-vaccination period, the cancelation of public events and gatherings was associated with 1.37 fewer deaths per 100,000 population [48]. In spite of the reduced effects of NPIs in a post-vaccine world, they can still play an important role. A study by Wang and coworkers reported a mathematical model for the effect of vaccines and NPIs. They reported that even full vaccination at the population level was not sufficient for herd immunity when the efficiency of vaccines was low, and as such, NPIs were still indispensable [49]. A systematic review by Brito and coworkers reported that some less disruptive NPIs, such as mask wearing requirements, were also effective [50]. It is worth noting, then, that while vaccinations are the most effective tool in reducing cases and deaths, NPIs can still provide a useful safety net [51,52].

### 4.3. Strengths and Limitations

The main strength of this study is the use of data from 164 different countries, which allows us to have a global picture of variation in vaccines and COVID-19 case fatality. We have also taken into account relevant confounding variables. The ecological nature of our study is its main limitation. We cannot establish a causal relationship between our variables of interest, and there is a possibility of confounding. Although our study cannot show a causal link, it identifies an association between higher vaccination coverage and lower mortality. Furthermore, the global nature of the data means the potential for discrepancies between case data and death data. For example, health-seeking behavior may vary globally [53], leading to a difference between reported cases and actual cases, as those with mild symptoms may not be tested. There may also be variations in how a COVID-19 death is defined [54]. Finally, our study could not account for the availability of different vaccines in countries or variations in the effectiveness of the various available vaccines.

### 4.4. Mechanism

The observed effects seen in more highly vaccinated populations may be related to secondary benefits from the vaccines. It has been reported that vaccines reduce overall infections and the severity of breakthrough infections [55]. This has been documented to occur with COVID-19 variants of concern [42]. This global analysis is consistent with other results, such as Haas and colleagues’ study of the effects of mass vaccination in Israel [56] and Bernal’s study of older adults in England [57]. A study in Scotland also showed that even a single dose lessened the risk of hospital admission [58]. Hence, together with other studies, this analysis adds valuable evidence to the positive effects of high rates of COVID-19 vaccination in preventing deaths and unfavorable outcomes.

Policies of vaccine rollout likely also contribute to the observed benefits of high vaccination coverage. These policies have generally prioritized vulnerable and high-risk populations, such as the elderly and healthcare workers [59]. The studies mentioned above have also reported the impact of a highly vaccinated population in improving health and protecting vulnerable groups, including a decrease in hospitalizations reported by Tartof et al. with a cohort of over 3 million individuals [18]. Our results also support this assertion since those infected are likely to belong to low-risk groups and experience severe disease or a fatality. It has been well documented that those with comorbidities and generally worse underlying health are at a higher risk for severe COVID-19 illness and death [5].

### 4.5. Implications

Our ecological study points to an association between lower mortality and higher vaccination rates but cannot establish a causal link. Further research, such as detailed country-level research, should examine the features of a successful COVID-19 vaccine implementation program to help identify the correlation between high vaccination rates and lower mortality at the country level. Studies similar to ours, which demonstrate an association, are the first step. Next, policy efforts must be directed towards increasing vaccination rates, helping shape public discourse around vaccination, combating misinformation campaigns [11], and reaching cultural and socioeconomic groups with higher vaccine hesitancy or less access to care [9].

Despite the potential and recognized benefits of increased vaccination coverage, there is still uneven vaccine distribution around the world [44], with a stark contrast between higher and lower-income countries, which is also seen in our results. Vaccine hesitancy is another factor impacting vaccine rollout in many countries [60,61,62]. Therefore, efforts to address the concerns of those who are hesitant and to correct misinformation, particularly on social media, must be implemented alongside efforts to increase global vaccine distribution.

## 5. Conclusions

There is considerable global variation in the percentage of fully vaccinated individuals in a country and COVID-19 mortality—there is a tendency for more vaccinations and fewer deaths per 1000 cases with increasing country income levels. An even larger effect was observed for booster doses, and similar trends were found within each income group. The potential benefits of vaccinating a large proportion of the population include preventing the emergence of further variants by reducing COVID-19 transmission and potentially reducing COVID-19 case mortality. Our findings underline the need for further global intensification of COVID-19 vaccine rollout, particularly in low-income countries, and the concomitant need to work towards equal access to vaccinations worldwide.

## Figures and Tables

**Table 1:** Sample characteristics.

	All Countries			High Income			Upper-Middle Income			Lower-Middle Income			Low Income		
	Median	IQR		Median	IQR		Median	IQR		Median	IQR		Median	IQR	
Deaths per 1000 cases (6 months)	15.68	9.84	25.87	12.26	5.42	19.40	15.63	11.51	26.17	11.96	48.03	24.89	22.48	11.96	48.03
Deaths per 1000 cases (12 months)	11.96	6.08	20.63	6.37	3.70	13.63	12.74	8.76	25.20	9.54	34.79	19.41	21.81	9.54	34.79
Percent of fully vaccinated people (6 months)	17.27	4.50	36.72	39.25	29.49	48.94	17.47	10.61	23.15	0.50	2.90	17.27	1.19	0.50	2.90
Percent of fully vaccinated people (12 months)	46.96	23.07	67.95	70.05	63.90	77.06	46.69	37.39	62.85	5.15	17.48	51.75	10.68	5.15	17.48
Percent of people receiving a boost dose (12 months)	18.85	5.56	36.21	36.21	23.43	46.01	10.80	6.15	20.63	0.05	0.82	13.41	0.29	0.05	0.82
GDP per capita (USD)	13,013	4877	33,084	43,153	33,084	57,807	14,850	11,601	18,833	1297	2339	8390	1913	1297	2339
Median age *	30.15	9.16		39.71	4.86		31.37	6.26		2.06			18.89	2.06	
Prevalence of obesity among adults *	18.46	9.50		24.97	6.39		22.67	6.40		5.47			7.72	5.47	
Age-standardized NCD mortality rate (per 100,000) *	543.98	172.55		396.36	124.23		540.99	141.94		113.06			665.90	113.06	

* Mean and Standard Deviation. IQR = Interquartile range; GDP = Gross Domestic Product; USD = United States Dollars; NCD = Non-Communicable Diseases.

**Table 2:** Association between vaccination and death case ratio.

Sample	All Countries	High Income	Upper-Middle Income	Lower-Middle Income	Low Income
	**Deaths per 1000 cases (6 months)**				
Percent of fully vaccinated people (6 months)	−0.0160 ***	−0.0133 *	−0.0145 *	−0.0188 *	−0.0193
	(0.00415)	(0.00738)	(0.00827)	(0.0101)	(0.0404)
Percent of fully vaccinated people (6 months) ^a^	−0.0181 ***	−0.0108	−0.0163	−0.0229 **	−0.0555
	(0.00545)	(0.00818)	(0.0125)	(0.0111)	(0.0616)
Observations	164	56	38	45	25
	**Deaths per 1000 cases (12 months)**				
Percent of fully vaccinated people (12 months)	−0.0176 ***	−0.0370 ***	0.00651	−0.0144 *	−0.0233 **
	(0.00316)	(0.0122)	(0.00593)	(0.00803)	(0.00898)
Percent of fully vaccinated people (12 months) ^a^	−0.0168 ***	−0.0239 *	0.00416	−0.0213 **	−0.0248 *
	(0.00503)	(0.0119)	(0.00721)	(0.00993)	(0.0138)
Observations	161	53	38	47	23
Percent of people receiving a boost dose (12 months)	−0.0310 ***	−0.0385 ***	−0.000922	−0.0300 *	−0.0336
	(0.00566)	(0.00896)	(0.0109)	(0.0167)	(0.0187)
Percent of people receiving a boost dose (12 months) ^a^	−0.0331 ***	−0.0305 ***	−0.00536	−0.0384 *	−0.0159
	(0.00873)	(0.0111)	(0.0148)	(0.0218)	(0.0368)
Observations	105	45	26	25	9

Estimated coefficients are based on ordinary least squares regressions with the log-transformed outcome and heteroscedasticity-robust SEs in parenthesis. Missing values on the predictor were imputed using Stata’s mi impute algorithm. * *p* values < 0.10; ** *p* value < 0.05; *** *p* value < 0.01. a = Adjusted for Gross Domestic Product per capita, median age of the population, the prevalence of obesity among adults, and age-standardized NCD mortality rate (per 100,000).

## Data Availability

This study used only freely available data.
